# Determination of minimum inhibitory and lethal concentrations of two lactic acid bacteria *Lactiplantibacillus plantarum* reveals strain-specific antibiotic resistance

**DOI:** 10.17912/micropub.biology.001615

**Published:** 2025-08-16

**Authors:** Maria V. Mendoza-Dasilva, Lorraine Rodriguez-Rivera, Imilce A. Rodriguez-Fernandez

**Affiliations:** 1 Biology, University of Puerto Rico at Río Piedras, San Juan, San Juan, Puerto Rico; 2 Natural Sciences, InterAmerican University of Puerto Rico, San Juan Campus, Puerto Rico

## Abstract

Lactic acid bacteria (LAB) are widely used as probiotics and in fermented foods, yet their antibiotic resistance profiles remain under-characterized. This study evaluates the antibiotic resistance profiles of two *Lactiplantibacillus plantarum* ( *L. plantarum* ) strains, LpWF (isolated from *Drosophila melanogaster* ) and Lp39 (from cabbage), using broth microdilution methods. Minimum inhibitory concentrations (MICs) and minimum lethal concentrations (MLCs) were determined for six antibiotics. Strain-specific differences were found in MICs for kanamycin, neomycin, and geneticin, while ampicillin and chloramphenicol MICs were similar. Both strains exhibited intrinsic resistance to vancomycin. These findings contribute to efforts to characterize the LAB resistome and inform probiotic strain selection.

**Figure 1. Minimum Inhibitory Concentration (MIC) and Minimal Lethal Concentration (MLC) results of six antibiotics tested against three bacterial strains.**
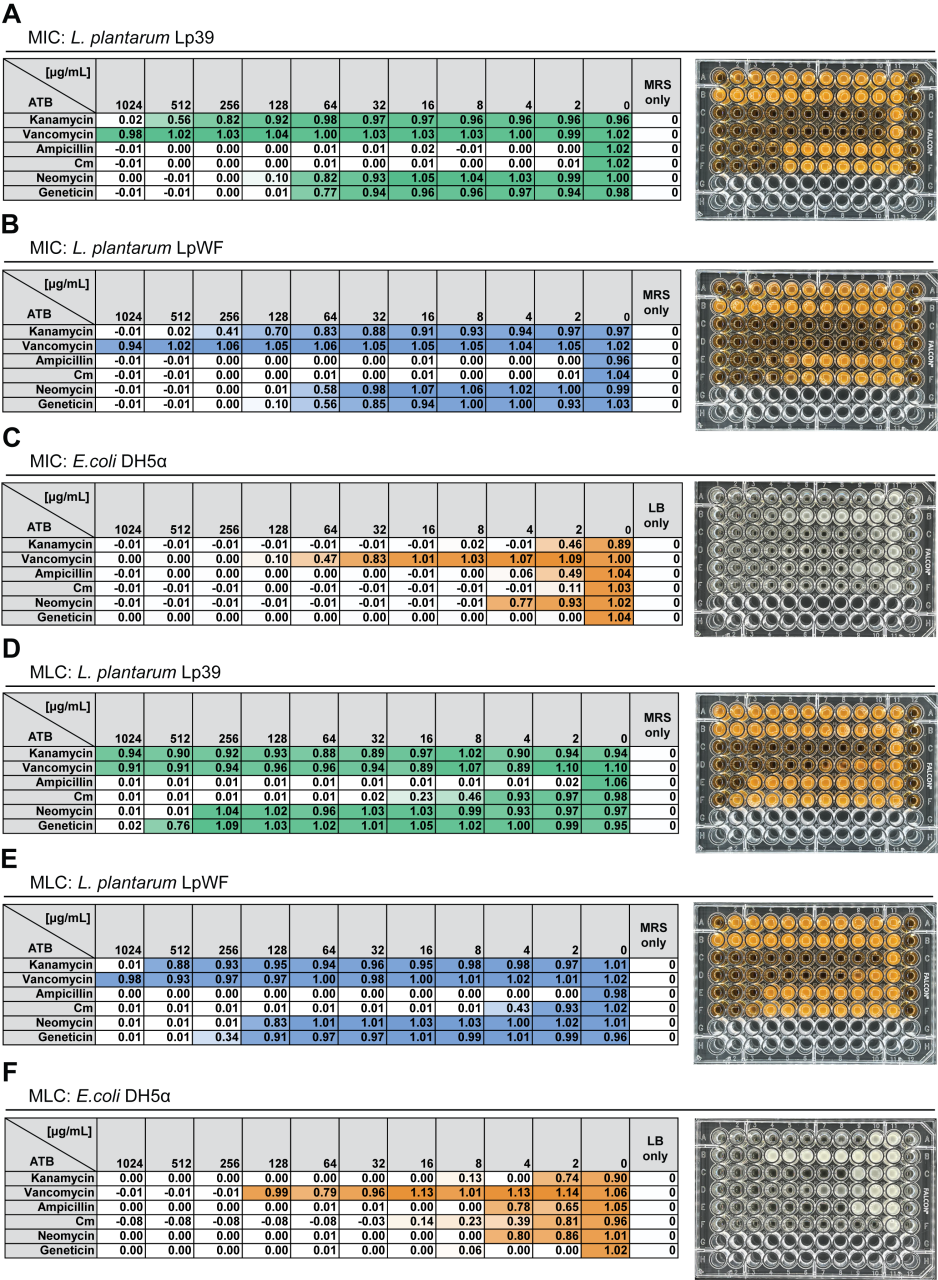
(A, D) MIC and MLC results for *Lactiplantibacillus plantarum* Lp39 (ATCC 14917, isolated from cabbage); (B, E) MIC and MLC results for *L. plantarum* LpWF (isolated from a wild *Drosophila* ); (C, F) MIC and MLC results for *Escherichia coli* DH5α. To allow direct comparison of bacterial growth across experiments, OD₆₀₀ values were normalized to bacterial growth OD₆₀₀ values were first background-corrected by subtracting the OD₆₀₀ of media-only controls, then normalized to the average OD₆₀₀ of bacterial growth in MRS broth without antibiotics. *Abbreviations:* ATB, antibiotics; Cm, chloramphenicol. *Colors* : Darker colors indicate higher growth, while lighter colors indicate lower growth. Each right-hand panel displays the corresponding 96-well plate image, where increased turbidity indicates greater bacterial growth.

## Description

Lactic acid bacteria (LAB), including members of the genus *Lactiplantibacillus* , play a central role in food fermentation, where many are part of the gut microbiota and are commonly used as probiotics due to their health-promoting properties (Hill et al. 2014; Zheng et al. 2020). As LABs are increasingly incorporated into dietary supplements, biotechnological applications and used for experimental design to understand host-microbe interactions, there is a growing need to assess their antibiotic resistance profiles to ensure biosafety and prevent horizontal gene transfer of antibiotic resistance genes (ARGs) (FAO/WHO 2001; Kim and Cha 2021)

*Lactiplantibacillus plantarum* , a genetically diverse LAB species, can be isolated from both plant- and animal-associated environments (Martino et al. 2016). While generally regarded as safe (GRAS) (US FDA), some strains have been shown to carry intrinsic or acquired antibiotic resistance, including resistance to vancomycin and aminoglycosides (Ferain et al. 1996; Elkins and Mullis 2004; Deghorain et al. 2007; Klarin et al. 2019; Tuerhong et al. 202). However, resistance patterns can vary between strains, and little is known about how ecological origin or niche may influence these profiles.

In this study, we compared two *L. plantarum* strains: LpWF, isolated from the gut of *Drosophila melanogaster* (Obadia et al. 2017), and Lp39 (ATCC 14917), isolated from fermented cabbage. We evaluated their susceptibility to six commonly used antibiotics: kanamycin, vancomycin, ampicillin, chloramphenicol, neomycin, and geneticin (summarized in **Table 1** ). We determined minimum inhibitory concentrations (MICs) and minimum lethal concentrations (MLCs) using the broth microdilution method. We used *Escherichia coli* DH5α as a control ( **Figure 1** ).

The MICs are summarized in **Table 2** *.* Overall, both strains of *L. plantarum* exhibited varying degrees of susceptibility to the antibiotics tested. As expected, vancomycin exhibited the highest MIC values for Lp39 and LpWF, reaching a MIC of >1024 µg/mL. While *E. coli* was susceptible at 256 µg/ml. This consistently high vancomycin MIC value indicates an intrinsic resistance to this antibiotic for Lp39 and LpWF similar to other *L. plantarum* strains (Ferain et al. 1996; Elkins and Mullis 2004; Deghorain et al. 2007; Klarin et al. 2019; Campedelli et al. 2019).

In contrast, ampicillin showed similar susceptibility patterns for Lp39 and LpWF, indicating comparable sensitivity to the antibiotic, consistent with previously reported literature (Campedelli et al. 2019; Kwon et al. 2021). For chloramphenicol, both strains have MICs of 2 µg/mL, which are lower than those reported for other *L. plantarum* strains (Campedelli et al. 2019; Kwon et al. 2021). Both *L. plantarum* strains had similar MICs of 128 µg/mL for geneticin. For neomycin, we find that Lp39 had a MIC of 256 µg/mL and LpWF of 128 µg/mL. Vancomycin, kanamycin and geneticin were effective at inhibiting *E. coli* growth at lower concentrations compared to *L. plantarum* .

Kanamycin demonstrated differential effectiveness for the *L. plantarum* strains. Lp39 showed a higher MIC of 1024 µg/mL, while LpWF was susceptible at a lower MIC of 512 µg/mL. This suggests strain-specific mechanisms that warrant further studies. Our *L. plantarum* ATCC 14917 strain exhibited a higher MIC to kanamycin (>1024 µg/mL) compared to previously reported values in the literature, which indicated a MIC of 64 µg/mL (Kwon et al. 2021). This discrepancy may be due to differences in the strain tested (Q180 in the previous study), experimental conditions, strain handling, or potential adaptive changes accumulated during laboratory propagation.

As for Minimal Lethal Concentrations (MLCs) values, results showed strain-specific differences for kanamycin (Lp39: >1024 µg/mL; LpWF: > 512 µg/mL), ampicillin (Lp39: 4 µg/mL; LpWF: 2 µg/mL), chloramphenicol (Lp39: 32 µg/mL; LpWF: 8 µg/mL), Neomycin (Lp39: 512 µg/mL; LpWF: 256 µg/mL), and Geneticin (Lp39: >1024 µg/mL; LpWF: 512 µg/mL). These findings suggest that while most antibiotics inhibited bacterial growth as shown with MICs, they fail to act as bactericides. This is expected in the case of chloramphenicol, a known bacteriostatic agent. While only one replicate is shown in **Figure 1,** variability in the MLCs was observed across replicates for *L. plantarum* , particularly for chloramphenicol, neomycin, and geneticin. Such variation may be due to differences in the *L. plantarum* inoculum used, likely resulting from variability in bacterial growth during the MIC assays. However, the differences between the two *L. plantarum* strains were consistently observed, highlighting their distinct antibiotic susceptibility profiles.

Although both strains belong to the same genus and species, *Lactiplantibacillus plantarum* , they exhibit notable differences in susceptibility to kanamycin, highlighting the strain-level variability in antibiotic resistance within this species. These differences may arise from genetic or phenotypic factors, such as variations in antibiotic uptake and efflux mechanisms, ribosomal target modifications, or strain-specific antimicrobial resistance (AMR) genes (Campedelli et al. 2019). Previous bioinformatic analyses have shown that AMR genes are present in some *L. plantarum* strains and can vary among isolates, supporting the need for individualized resistance profiling (Ferain et al. 1996; Elkins and Mullis 2004; Deghorain et al. 2007; Klarin et al. 2019; Kwon et al. 2021). This variation underscores the importance of strain-specific susceptibility testing and suggests that both genetic and environmental factors may contribute to the development of distinct resistance profiles, even among strains of the same species (Tuerhong et al. 2024).

Both strains were phenotypically resistant to vancomycin, consistent with previously reported intrinsic resistance in LAB species. This is likely due to the presence of D-Ala-D-Lac termini in the peptidoglycan precursors rather than the acquisition of mobile resistance elements (Ferain et al. 1996; Deghorain et al. 2007).

Similarly, resistance to aminoglycosides such as kanamycin and neomycin appears to be intrinsic. This pattern is consistent with prior reports showing that *L. plantarum* strain WCFS1 and related LABs possess low membrane permeability, which limits aminoglycoside uptake and confers natural resistance (Elkins and Mullis 2004). Thus, the aminoglycoside resistance observed in Lp39 and LpWF is best attributed to this intrinsic mechanism rather than horizontal gene transfer or mobile genetic elements.

In addition to chromosomal differences, plasmid-encoded traits may also contribute to these strain-specific resistance profiles. Although plasmid profiles were not assessed in this study, *L. plantarum* strains are known to harbor plasmids encoding a wide array of stress-related functions. For example, strain WCFS1 carries plasmid pWCFS103, which encodes genes involved in redox metabolism and arsenate resistance (van Kranenburg et al., 2005). A broader analysis of 105 *L. plantarum* strains identified 395 plasmids, many encoding strain-specific genes associated with oxidative stress response and heavy metal resistance (Davray et al., 2023). While these functions are not canonical antibiotic resistance determinants, they may enhance cellular resilience and indirectly influence susceptibility to antibiotics such as kanamycin. Additionally, recent sequencing of the LpWF genome revealed five plasmids, including one carrying genes linked to *Drosophila* gut colonization not found in ATCC 8014 (Gutiérrez-García et al., 2024). All these studies highlight strain-specific adaptive functions potentially encoded by plasmids. Future comparative analysis of plasmid content in Lp39 and LpWF may help clarify their contribution to the MIC differences observed in this study.

In summary, our findings highlight the complex and strain-specific nature of antibiotic resistance in *L. plantarum* . Both Lp39 and LpWF exhibited intrinsic resistance to vancomycin. Notably, Lp39 showed resistance to kanamycin, while LpWF remained susceptible, though only at high concentrations. Both strains were susceptible to ampicillin and chloramphenicol. These findings also highlight the need for further research into the molecular mechanisms underlying strain-specific resistance in *L. plantarum* .

**Table 2** . Minimum inhibitory concentrations (MICs) of antibiotics for strain *L. plantarum* ATCC 14917 (Lp39), *L. plantarum* isolated from the gut of the wild fly (LpWF), and *E. coli* DH5α.

**Table:** 

	MIC (µg/ml)		
Antibiotic	*L. plantarum* (Lp39)	*L. plantarum* (LpWF)	*E. coli* (DH5α)
Kanamycin	1024	512	4
Vancomycin	>1024	>1024	256
Ampicillin	2	2	4
Chloramphenicol	2	2	2
Neomycin	256	128	8
Geneticin	128	128	2

## Methods

The minimum inhibitory concentration (MIC) of these 6 antibiotics was determined using broth microdilution methods according to the Clinical and Laboratory Standards Institute (CLSI; www.clsi.org)

Each antibiotic was prepared at an initial concentration of 1024 µg/mL and performed two-fold serial dilutions down to a concentration of 2 µg/mL in a 96-well plate, resulting in a range of concentrations. Each well in the plate contained 200 µL of total volume.

Bacterial cultures were prepared by growing the strains in their respective media for 24 hours in aerobic conditions. After growth, the bacterial cultures were adjusted to an optical density at 600 nm (OD₆₀₀) corresponding to 1x10 ^6^ colony-forming units (CFU)/mL, based on spectrophotometer readings. A 10 µL aliquot of each bacterial suspension was inoculated into each well containing the antibiotic dilutions, resulting in a final volume of 210 µL per well. The inoculated 96-well plates were incubated at 37°C for 24 hours. After incubation, we quantified bacterial growth by recording the absorbance at 600 nm using a Tecan plate reader. Each MIC and MLC assay was performed using three independent biological replicates, each with at least two technical replicates, for a total of n = 7 measurements per antibiotic per strain.

To determine MLCs, an aliquot of 10 ul from each well of the 96-well plate used for MIC determination was transferred into a new 96-well plate containing fresh culture broth (MRS or LB, depending on the strain tested). The plate was then incubated under the same conditions described above, and bacterial growth was assessed by measuring OD₆₀₀.

For each MIC/MLC assay, absorbance at OD₆₀₀ was measured for all wells after incubation. To correct for background, the OD₆₀₀ value of the corresponding media-only control (MRS without bacteria) was subtracted from each well. Subsequently, these background-corrected OD₆₀₀ values were normalized to the average OD₆₀₀ of the bacterial growth control wells containing MRS broth without antibiotics. This two-step normalization allowed for direct comparison of bacterial growth across different antibiotic concentrations and strains.

**Table 1. Classification and Spectrum of Selected Antibiotics ^1^**

**Table:** 

**Antibiotic**	**Type / MOA**	**Spectrum**
Kanamycin	Aminoglycoside / 30S ribosome inhibitor	Broad-spectrum (Gram-negative, some Gram-positive)
Vancomycin	Glycopeptide / Cell wall biosynthesis inhibitor	Narrow-spectrum (Primarily Gram-positive)
Ampicillin	β-lactam / Cell wall biosynthesis inhibitor	Broad-spectrum (Gram-positive, some Gram-negative)
Chloramphenicol	Amphenicol / 50S ribosome inhibitor	Broad-spectrum (Gram-positive and Gram-negative)
Neomycin	Aminoglycoside / 30S ribosome inhibitor	Broad-spectrum (Gram-negative, some Gram-positive)
Geneticin	Aminoglycoside / 30S ribosome inhibitor	Broad-spectrum (Primarily Gram-negative, some Gram-positive)

^1^ Based on (Murray et al. 2021)

## Reagents

This study used three bacterial strains: 1) *Lactiplantibacillus plantarum* isolated from the gut of wild-type *Drosophila melanogaster* flies (Obadia et al. 2017), kindly provided by Dr. William Ludington (Carnegie Science); 2) *Escherichia coli* DH5α kindly provided by Dr. Jose A. Rodriguez Martinez (University of Puerto Rico Rio Piedras) and 3) *Lactiplantibacillus plantarum* ATCC 14971. Both E. coli DH5α and *L. plantarum* Lp39 ATCC 14917 are commercially available.

We used the Lactobacilli De Man, Rogosa, and Sharpe (MRS) broth (BD Difco) for *L. plantarum* cultures and Luria Broth (LB) (Sigma-Aldrich) for *E. coli* cultures. All cultures were done in sterile 96-well plates clear flat-bottom and OD600 was analyzed using Tecan Plate Reader.

The antibiotics tested were kanamycin, vancomycin, ampicillin, chloramphenicol, neomycin, and geneticin (Gold Bio Technology). Antibiotic stock solutions were prepared at a concentration of 1024 μg/mL in sterile double-distilled water and 2-fold serially diluted in MRS or LB medium as required. Chloramphenicol stock solution was prepared at the same concentration but dissolved in 95% ethanol instead of water.
